# Spraying Fluorinated Silicon Oxide Nanoparticles on CuONPs@CF-PVDF Membrane: A Simple Method to Achieve Superhydrophobic Surfaces and High Flux in Direct Contact Membrane Distillation

**DOI:** 10.3390/polym14235164

**Published:** 2022-11-27

**Authors:** Zivka Lenac, César Saldías, Claudio A. Terraza, Angel Leiva, Joachim Koschikowski, Daniel Winter, Alain Tundidor-Camba, Rudy Martin-Trasanco

**Affiliations:** 1 Research Laboratory for Organic Polymers (RLOP), Faculty of Chemistry and of Pharmacy, Pontificia Universidad Católica de Chile, Santiago 7820436, Chile; 2 Department of Physical Chemistry, Faculty of Chemistry and of Pharmacy, Pontificia Universidad Católica de Chile, Santiago 7820436, Chile; 3 UC Energy Research Center, Pontificia Universidad Católica de Chile, Santiago 7820436, Chile; 4 Fraunhofer Institute for Solar Energy Systems (ISE), 79110 Freiburg, Germany; 5 Departamento de Química, Universidad Tecnológica Metropolitana, Las Palmeras 3360, Santiago 8940577, Chile

**Keywords:** superhydrophobic surfaces, PVDF membranes, direct contact membrane distillation, fluorinated capped silicon oxide nanoparticles

## Abstract

Desalinization of seawater can be achieved by membrane distillation techniques (MD). In MD, the membranes should be resistant to fouling, robust for extended operating time, and preferably provide a superhydrophobic surface. In this work, we report the preparation and characterization of a robust and superhydrophobic polyvinylidene fluoride membrane containing fluoroalkyl-capped CuONPs (CuONPs@CF) in the inner and fluorinated capped silicon oxide nanoparticles (SiO_2_NPs@CF) on its surface. SiO_2_NPs@CF with a mean diameter of 225 ± 20 nm were prepared by the sol method using 1H,1H,2H,2H-perfluorodecyltriethoxysilane as a capping agent. Surface modification of the membrane was carried out by spraying SiO_2_NPs@CF (5% wt.) dispersed in a mixture of dimethyl formamide (DMF) and ethanol (EtOH) at different DMF/EtOH % *v*/*v* ratios (0, 5, 10, 20, and 50). While ethanol dispersed the nanoparticles in the spraying solution, DMF dissolved the PVDF on the surface and retained the sprayed nanoparticles. According to SEM micrographs and water contact angle measurements, the best results were achieved by depositing the nanoparticles at 10% *v*/*v* of DMF/EtOH. Under these conditions, a SiO_2_NPs covered surface was observed with a water contact angle of 168.5°. The water contact angle was retained after the sonication of the membrane, indicating that the modification was successfully achieved. The membrane with SiO_2_NPs@CF showed a flux of 14.3 kg(m^2^·h)^−1^, 3.4 times higher than the unmodified version. The method presented herein avoids the complicated modification procedure offered by chemical step modification and, due to its simplicity, could be scalable to a commercial membrane.

## 1. Introduction

By 2050 humanity will be facing one of its greatest crises, relating to water. On the Earth’s surface, 3% of water is considered consumable, but only 0.9% is accessible [1]. Fortunately, the oceans store 97% of water, and in the future, this water source should be accessible for human consumption by membrane, thermal, or hybrid desalinization processes [2]. Among these, membrane distillation (MD) has advantages over thermal techniques through its lower energy demands (low-grade energy and renewable energy sources), higher efficiency (100% rejection of nonvolatile solute), and reduced installation-space requirements [2,3,4].

In MD techniques, a membrane allows only the passage of water vapor through pores, which is driven by a vapor pressure gradient and avoids condensation in the inner material [4]. Polymeric and ceramic membranes have been continuously investigated to improve their properties and optimize their performance. Although ceramic membranes can operate under harsh conditions, their cost make them less favorable than polymeric membranes [5,6]. In addition to being cheaper, the latter can be modified and subjected to novel formulations by preparation via non-solvent induced phase separation methods (NIPS). This method allows composited membranes to be prepared with desired properties. Polypropylene (PP), polyethylene (PE), and polyvinylidene fluoride (PVDF) are among the commercial polymers most widely used for this purpose [1]. PVDF has high selectivity and a high tolerance to chemicals. Additionally, it is soluble in aprotic solvents (DMF, DMAc, NMP, and DMSO), allowing a casting solution method for preparing electrospun nanofiber membranes, flat sheet, or hollow fiber membranes via NIPS. Therefore, many composite membranes containing metal (Ag), metal-oxide nanoparticles (SiO_2_, CuO, TiO_2_, ZnO, Al_2_O_3_, and ZrO), carbon nanomaterials, multiwalled carbon nanotubes (MwCNTs), graphene oxide (GrO), and activated carbon have been already prepared using this polymer [7,8,9,10].

Hydrophobic or superhydrophobic properties of either the surface or inner membranes are a mandatory condition, because they must retain the liquid phase and allow only the vapor to pass through. Superhydrophobic membranes which have a water contact angle (WCA) higher than 150° and sliding angles less than 10° are achieved by either increasing the surface roughness or decreasing the surface energy via chemical modification [10,11]. High surface roughness is achieved by changing the composition of the coagulation bath with a coprecipitating agent (i.e., methanol or isopropyl alcohol). Phase inversion, interfacial polymerization, cross-linking, and chemical grafting are common methods to prepare the chemically modified surface [12,13]. Through assembling hierarchical structures on the membrane surface (self-assembly or layer-by-layer), both features (high roughness and low surface energy) can be attained, and highly efficient and robust membranes have been prepared [14,15].

The surface modification of PVDF membrane with hydrophobic SiO_2_ nanoparticles is one of the most versatile methods to achieve superhydrophobic and antifouling surfaces [16,17]. Zhang et al. prepared a micro/nanocomposite hierarchical membrane by coating the PVDF surface with SiO_2_NPs and grafting 1H,1H,2H,2H–perfluoroocyltrichlorosilane onto SiO_2_NPs [16]. Omniphobic PVDF membranes were prepared by Boo et al. in a layer-by-layer fashion by enriching the membrane surface with hydroxyl groups to graft (3-aminopropyl) trietoxysilane. SiO_2_NPs were assembled onto the hydroxyl layer by electrostatic interactions and further capped with perfluorodecyltrichlorosilane via vapor-phase salinization [18]. Recently, Wai et al. similarly prepared a hierarchical superhydrophobic PVDF composite membrane by firstly coating the PVDF surface with a layer of poly(catechol/polyamine). Onto this layer, silver nanoparticles were synthesized in situ, capped with 1H,1H,2H,2H-perfluorodecanethiol, and later cured with polydimethylsiloxane [14]. Superhydrophobic composited electrospun PVDF nanofiber membrane with high biofouling resistance was prepared by Nthunya et al., and its superhydrophic character was achieved by electrospinning the casting solution containing SiO_2_NPs coated with octadecyltrimethoxysilane, *N*-octadecyltrichlorosilane, and chlorodimethyloctadecyl silane. Biofouling resistance was achieved by coating the resulting membrane with carboxylated multi-walled carbon nanotubes and silver nanoparticles [7]. Although the methods mentioned above have helped to prepare superhydrophobic surfaces, industrial scaling remains a limitation.

Spray deposition is a simple and versatile industrial process that can be applied to prepare superhydrophobic surfaces. A well-covered surface can be obtained by controlling the condition of the spray application and the concentration of the precursor. Zhang et al. prepared a superhydrophobic surface by spraying a mixture of polydimethylsiloxane and hydrophobic-fumed SiO_2_NPs onto PVDF flat sheet membranes, which showed an increase in WCA, liquid entry pressure, and higher salt rejection at long-term operation in comparison to the unmodified membrane. Nevertheless, the flux of the modified membrane was lower than that of the unmodified [19].

Recently, we reported the preparation of PVDF composite membranes containing hydrophobically capped copper oxide nanoparticles (CuONPs) supported on non-woven polyester fabric (NWPET), and their performance in direct contact membrane distillation (DCMD). The prepared membranes showed a 100% salt rejection and fluxes of 1.8, 2.7, and 3.9 kg(m^2^·h)^−1^ for naked, alkyl (CuONps@CH), and fluoroalkyl (CuONPs@CF) capped CuONPs, respectively [4]. We hypothesize that the low flux values achieved were due to the moderate contact angles obtained (WCA 80–95°), despite the presence of the hydrophobically capped copper nanoparticles.

In the present work, we prepared CuONPs@CF-PVDF composite films supported on NWPET as substrate, and then sprayed synthesized fluoroalkyl-capped SiO_2_NPs onto the membrane surface. Fluorination of SiO_2_NPs should enhance the hydrophobicity of the membrane surface not only because of the increase in surface roughness, according to the Cassie–Baxter state, but also due to the decrease of the surface energy driven by the fluorinated alkyl chains on the surface of SiO_2_NPs@CF. As a result, a superhydrophobic membrane (CuONPs@CF-PVDF\SiO_2_NPs@CF) was obtained with higher WCA and flux values than our previous work. Additionally, the membrane retained 100% salt and was reusable after 24 h of workup.

## 2. Materials and Method

### 2.1. Materials

Non-woven polyester fabric (NWPET) was purchased from Importadora Dilaco S.A. (Santiago, Chile). Copper oxide nanoparticles (CuONPs, diameter < 50 nm), poly(vinylidene fluoride) (PVDF, average molecular weight of ~180 kDa by GPC, average Mn ~71 kDa, beads or pellets), *N, N*-dimethylformamide (DMF, ≥ 99.8%), ammonia (NH_4_OH, 25%), ethanol (≥99%), n-octanethiol (≥98.5%), 1H,1H,2H,2H-perfluorodecanethiol (97%), tetraethylorthosilicate (TEOS, > 98%), and 1H,1H,2H,2H-perfluorodecyltriethoxysilane (PFDSi, > 97%) were purchased from Sigma-Aldrich (Milwaukee, WI, USA) and were used without further purification. Fluoroalkyl-capped CuONPs were prepared as reported elsewhere [4].

### 2.2. Preparation of SiO_2_NPs@CF Nanoparticles

Hydrophobic SiO_2_NPs were obtained by the reaction of TEOS and PFDSi in a basic medium accordingly to the method reported by Wang et al. [20]. In brief, TEOS (5 mL, 22.4 mmol) was dissolved in methanol (25 mL), and PFDSi (1 mL, 2.24 mmol) was added to the solution. Then, an ethanol solution (25 mL) containing ammonia solution (7 mL, 25%) was added, and the resulting mixture was stirred overnight at room temperature. The resulting dispersion was centrifugated (12,000 rpm), and the residue was washed with ethanol (×5). Finally, the solid was dried in a vacuum oven at 60 °C overnight.

### 2.3. Preparation and Surface Modification of PVDF Composited Membrane

According to our previous results, the best performance by the composited membrane was obtained from the preparation with CuONPs@CF at 10% wt. [4]. Therefore, this membrane was prepared and selected for surface modification with SiO_2_NPs@CF in triplicate. In brief, a solution of PVDF in DMF (20% wt./v) containing CuONPs@CF (10%w.t, CuONPs@CF-PVDF) was prepared and cast on NWPET. As a coagulation bath, the casted NWPET was gently dipped in water at 25 °C. The bath was replaced several times with freshly distilled water to remove the residual DMF. The formed membrane was left there for 24 h and then dried in an oven, at 50 °C, for 12 h.

SiO_2_NPs@CF (50 mg) were dispersed in a DMF/EtOH mixture (10 mL) at different *v*/*v*.% (0, 5, 10, 20 and 50% *v*/*v*). The dispersion was sonicated at 25 °C for 30 min in an ultrasonic bath, and poured into a hand sprayer to spread onto the CuONPs@CF-PVDF surface. The spraying process was conducted under the same conditions by depositing the SiO_2_NPs@CF dispersion (5 mL) from a fixed distance (40 cm) over the surface. After drying in an oven at 40 °C overnight, the resulting modified membrane was sonicated and washed with plenty of water to remove unattached SiO_2_NPs@CF.

### 2.4. Instrumentation

Infrared spectra were recorded on a Perkin-Elmer Spectrum-Two spectrometer (PerkinElmer Inc., Waltham, MA, USA) with a coupled universal attenuated total reflection (UATR) unit. Samples were placed over the diamond, pressed until reaching 30% of the total supported pressure, and scanned in the range 4000 to 500 cm^−1^ with a resolution of 1 cm^−1^. Electron microscopy characterization of the CuONPs@CF-PVDF\SiO_2_NPs@CF composited membrane was performed using a Zeiss model EVO MA 10 electron microscope (Carl Zeiss Pvt. Ltd., Oberkochen, Germany). The cross-section SEM micrographs were acquired by fracturing the membranes, using liquid nitrogen to freeze them, and applying a surgical scalpel to cut the NW-PET. The membranes were coated with gold using a Cressington-108 auto sputter coater (Carl Zeiss Pvt. Ltd., Oberkochen, Germany). The recorded SEM micrographs were measured and processed using the free ImageJ (version 1.46 J/Fiji) software package from the National Institute of Health, Bethesda, MD, USA [21]. Water contact angle (WCA) measurements were performed using the sessile drop technique, employing Dataphysics OCA 20 (DataPhysics, Filderstadt, Germany). A syringe connected to a capillary of Teflon (2 mm internal diameter) was used to place a water drop (10 µL) on the membrane surface. A camera, coupled with the equipment, was employed to acquire images of the deposited drops, and the WCA values were measured through computational processing of the drop profile. Five measurements were taken along the membrane surface, and the averaged WCA values were calculated.

### 2.5. Membrane Porosity Measurement

Membrane porosity was determined by the gravimetric method reported elsewhere [4]. The prepared membranes were fully immersed for 24 h in a sealed flask containing *n*-butanol. After superficially drying by gently pressing between two pieces of filter paper, the membranes were weighed ($w_{1}$) and later weighed again ($w_{2}$) after drying in an oven at 50 °C. The membrane porosity (ε) was calculated as per Equation (1):

(1) $$\varepsilon \left(\%\right)=\frac{\left(w_{1}-w_{2}\right)\rho_{2}}{\rho_{2}w_{2}+\left(\rho_{1}-\rho_{2}\right)w_{2}}\times 100$$

where $\rho_{1}$ and $\rho_{2}$ are the density at 25 °C of PVDF (1.78 g·cm^−3^) and *n*-butanol (0.810 g·cm^−3^), respectively.

### 2.6. Membrane Performance

**Scheme 1:**
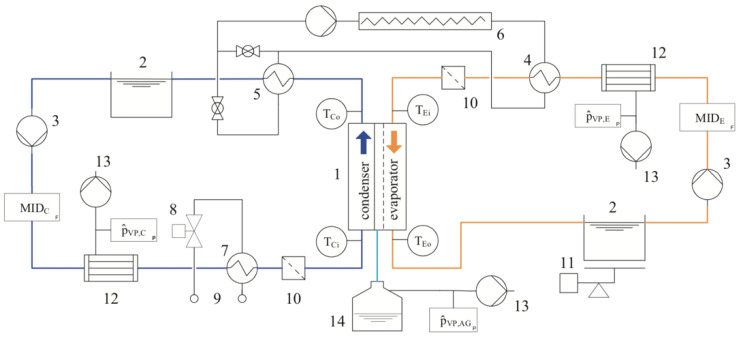
Hydraulic layout of the experimental setup; 1: membrane test cell; 2: feed water tank; 3: feed pump; 4: heat exchanger; 5: auxiliary heat exchanger; 6: electric heater; 7: heat exchanger; 8: motorized valve; 9: laboratory cooling circuit; 10: filter; 11: electronic balance; 12: deaeration module; 13: vacuum pump; 14: permeate tank [22].

The performance of the membrane in the DCMD setup was determined in a membrane distillation unit, as represented in Scheme 1. Flux values were measured in triplicate at three different feed and permeation temperatures (T*_f_* − T*_p_* = (64–56) °C, (64–44) °C, and (80–50) °C). The feed solution consisted of a 0.1 wt% aqueous sodium chloride solution. The flux was measured according to Equation (2):

(2) $$J=\frac{w}{A\cdot t}$$

where *J* is flux in kg(m^2^·h)^−1^, and *w* (kg), A = 0.0375 m^2^, and *t* (h) are the weight of the permeate collected, effective membrane area, and filtration time, respectively. The salt retention was checked by measuring the conductivity on both sides of the membrane.

## 3. Results and Discussion 

### 3.1. SiO_2_NPs@CF Synthesis and Characterization

**Scheme 2:**
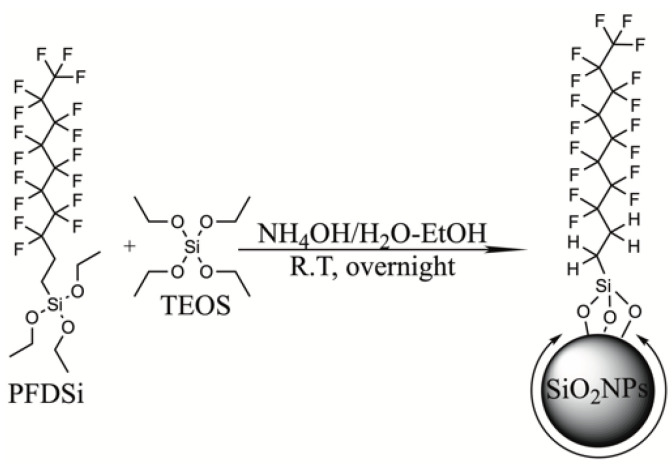
Synthesis of SiO_2_NPs@CF.

Hydrophobic SiO_2_NPs@CF were obtained in a one-pot reaction by the co-hydrolysis and condensation of TEOS and PFDSi. The latter limits the growth of nanoparticles to produce a narrow size distribution and confers hydrophobicity to their surface (Scheme 2).

**Figure 1:**
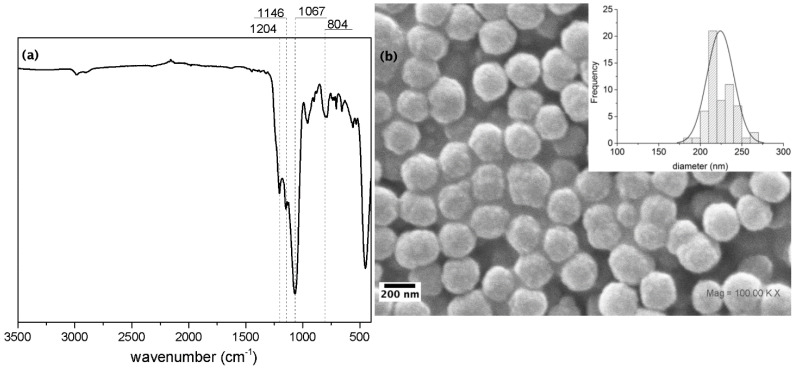
(**a**) ATR-FTIR and (**b**) representative SEM micrograph of SiO_2_NPs@CF, with (inset) the corresponding histogram.

The SiO_2_NPs@CF were characterized by ATR-FTIR spectroscopy. In the IR spectrum (Figure 1a), two intense bands corresponding to symmetric and antisymmetric -C-F stretching in the PFDSi moiety were recorded at 1146 cm^−1^ $\left(\nu_{CF}^{s}\right)$ and 1204 cm^−1^$\;(\nu_{CF}^{as})$, respectively. Similarly, bands from the stretching modes of -C-H groups were observed at 2892 cm^−1^ ($\nu_{CH}^{s}$) and 2980 cm^−1^ ($\nu_{CH}^{as}$). Additionally, the spectrum showed two other bands at 810 cm^−1^ and 1067 cm^−1^ corresponding to $\nu_{SiOSi}^{s}\;$ and $\nu_{SiOSi}^{as}$. These two later bands indicated the presence of Si-O-Si linkage, and therefore the formation of the SiO_2_ core. Scanning electron microscopy corroborated the formation of SiO_2_NPs@CF. Spherical nanoparticles with a mean diameter of 225 ± 20 nm were obtained, as shown in Figure 1b.

### 3.2. Membrane Preparation and Characterization

**Figure 2:**
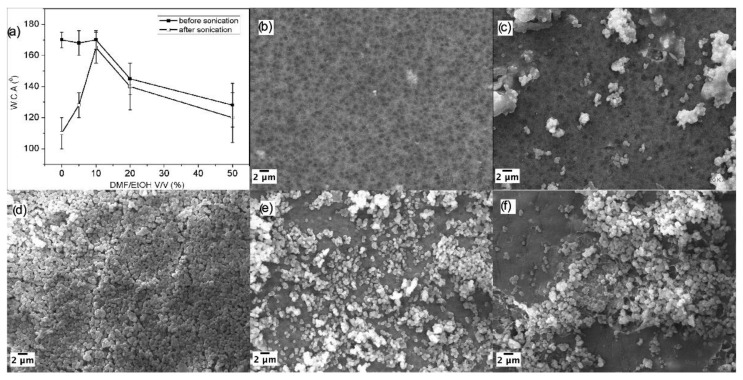
(**a**) Influence of volume ratio of DMF/EtOH (*v*/*v*%) on the WCA values of the modified membrane, before (filled square) and after (open square) sonication. SEM micrographs after sonication of the membrane surface sprayed with the solution containing DMF at (**b**) 0%, (**c**) 5%, (**d**) 10%, (**e**) 20% and (**f**) 50% *v*/*v*.

Membranes were prepared via the NIPS method, and the SiO_2_NPs@CF dispersed in a DMF/EtOH mixture were sprayed onto the surface. Different volume ratios of DMF/EtOH (0, 5, 10, 20, and 50% *v*/*v*) were utilized, to ensure the adhesion of the SiO_2_NPs@CF on the surface. DMF partially dissolves the PVDF at the surface, while EtOH improves the dispersion of the nanoparticles in the spraying solution. Figure 2a shows the WCA values of the membranes at different DMF/EtOH dispersant ratios.

As can be noted from Figure 2a, at a low DMF/EtOH ratio (≤ 5% *v*/*v*), membranes showed superhydrophobic surfaces, but after sonication WCA decreased by 50°. However, at DMF/EtOH ratios higher than 10% *v*/*v*, sonication did not affect the hydrophobicity of the membranes. Nevertheless, the WCA was lower than 150°, so the membranes do not fulfill superhydrophobic conditions. Regardless, for the 10% mixture, the WCA value was higher than 160°. This value was retained after sonication, indicating an increased adhesion of nanoparticles and therefore a robust surface modification.

At a low DMF/EtOH ratio, SiO_2_NPs@CF are weakly adsorbed on the membrane, and the cavitation energy of sonication is enough to remove them from the surface. This fact is reflected in Figure 2b, where a bare membrane surface is observed in the absence of DMF in the spraying solution. At 5% *v*/*v* of DMF composition (Figure 2c), some populations of nanoparticles were present, but they scarcely covered the surface. These results are expected since PVDF is barely soluble in ethanol, and no nanoparticle retention occurs. On the other hand, at high solvent ratios, DMF dissolves the PVDF on the surface, and the SiO_2_NPs@CF are retained after drying. A homogeneous surface covering is observed in Figure 2d, achieved by spraying the nanoparticles using a solution with a DMF/EtOH ratio of 10% *v*/*v*. Increasing the DMF ratio to 20% *v*/*v* and 50% *v*/*v*, the nanoparticles appeared to be aggregated on the surface rather than well dispersed. A high content of DMF decreases the dispersibility of the SiO_2_NPs@CF, which form aggregates and result in non-homogeneously deposits upon spraying (Figure 2e,f). Based on the results described above, the membrane prepared with 10% of DMF in the spraying solution was selected for further characterization and study of performance. 

**Figure 3:**
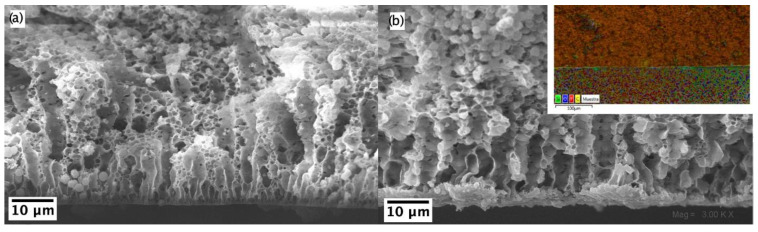
Cross-section SEM micrograph of (**a**) unmodified and (**b**) modified membrane surface. Inset of (**b**) shows the surface EDS mapping.

It is important that the addition of DMF to the spraying mixture does not affect the porosity or morphology of pores in the prepared membranes. Both features are crucial in membranes used in MD. Finger-like pores beneath the surface lead to a capillary effect that increases the vapor pressure of the liquid and favors flux into the membrane. In contrast, porosity lowers the thermal conductivity and therefore increases the efficiency of the process. Figure 3 displays the cross-section SEM micrograph of the unmodified (Figure 3a) and modified (Figure 3b) membrane prepared at 10% *v*/*v* DMF.

As noted, the unmodified membrane (Figure 3a) presents a flat surface and a typical finger-like pore structure, extending to the inner membrane. The modified membrane (Figure 3b) shows a thin layer of SiO_2_NPs@CF deposited on its surface, and finger-like pores beneath this, as in the unmodified membrane. The EDS surface map (Figure 3b. inset) shows that the surface is covered by silicon element (green color) from the SiO_2_NPs@CF. This result indicates that adding DMF and dissolving PVDF on the surface did not affect the morphology of the pores in the membrane. Additionally, in the SEM micrograph we determined the porosity of the membrane via gravimetric methods using *n*-butanol as a wetting solvent. The mean value of five measurements showed that the membrane had 63 ± 4% of porosity. This value is similar to that in the membrane prepared without SiO_2_NPs@CF nanoparticles (65%), and indicates that the surface modification did not affect the integrity of the whole membrane. 

### 3.3. DCMD Performance of the Modified Membrane

**Figure 4:**
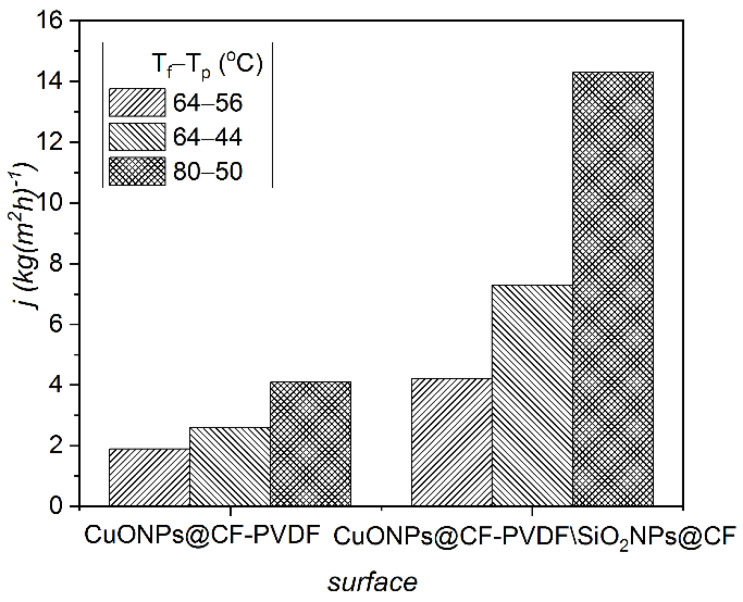
Performance of membranes at different *∆T*.

According to WCA, the cross-section SEM micrographs, and the porosity results, the membranes should theoretically be able to distill water. Nevertheless, it is important to consider that the adhesion of SiO_2_NPs@CF upon spraying with 10% *v*/*v* DMF-EtOH might seal the pores on the membrane surface and disrupt the connection with the finger-like pores beneath. Therefore, we studied the performance of the membrane in the DCMD setup at three different *∆T = T_feed_ − T_permeated_* temperature values ((64–56) °C, (64–44) °C, and (80–50) °C) Figure 4).

As shown in Figure 4, at the three DT temperatures assayed, the fluxes of the modified membranes increased for the same DT values 2.2, 2.8, and 3.4 times in comparison with the unmodified membranes. Therefore, from these results, we can conclude that the modification of the membrane surface does not close its pores, and that the presence of SiO_2_NPs@CF increases the hydrophobicity and hence the attained flux. Contrary to reports in other works, in which a decrease in flux was observed with modification of the surface, in our case, the modified membrane led to higher flux [7,16,18]. We can suggest that hydrophobic SiO_2_NPs@CF, instead of becoming an additional barrier to mass transfer, by increasing the hydrophobicity of the membrane increases the vapor pressure at the entrance of the surface pores and favors increased flux. As expected, higher flux was achieved at DT = 30 °C (14.3 kg(m^2^h) ^−1^). It should be highlighted that this flux is similar to the values obtained in other works in which membranes modified with SiO_2_NPs have employed (Table 1).

## 4. Conclusions

Hydrophobically capped membrane with a superhydrophobic surface (WCA higher than 160°) was prepared by embedding SiO_2_NPs@CF on the surface of the substrate (CuONPs@CF-PVDF). A uniform layer of nanoparticles was obtained on the membrane surface by spraying 5% wt. of these in a mixture of DMF/EtOH (10% *v*/*v*). The spraying of nanoparticles did not disrupt porosity or pore entry on the membrane surface, similar to the unmodified membrane. However, the modified membranes showed higher flux values than the unmodified ones. Avoiding the complex preparation procedures and purification steps reported by other authors, which represent drawbacks to upscaling for feasible application, spray deposition successfully provided a simple and versatile method to prepare superhydrophobic surfaces.

## Figures and Tables

**Table 1:** Comparison of flux results with other membranes modified with SiO_2_NPs.

Membrane	T_F–_T_p_ °C	Porosity (%)	WCA (^0^)	*J* (kg(m^2^h)^−1^)	Ref.
PVDF-LiCl\SiO_2_NPs-PFOTS	80–25	66.1	161.5	12.0	[16]
PVDF\SiNPs	60–20	-	>150	13.6	[18]
PVDF\SiO_2_NPs-fMWCN-AgNPs	60–20	-	159.0	16.0	[7]
CuONPs@CF-PVDF	80–50	62.0	95.60	4.10	t.w
CuONPs@CF-PVDF\SiO_2_NPs@CF	80–50	63.0	164.6	14.3	t.w

t.w.: This work.

## Data Availability

Not applicable.
